# Cost of acute renal replacement therapy in the intensive care unit: results from The Beginning and Ending Supportive Therapy for the Kidney (BEST Kidney) Study

**DOI:** 10.1186/cc8933

**Published:** 2010-03-26

**Authors:** Nattachai Srisawat, Loredo Lawsin, Shigehiko Uchino, Rinaldo Bellomo, John A Kellum

**Affiliations:** 1 The CRISMA (Clinical Research, Investigation, and Systems Modeling of Acute Illness) Laboratory, Department of Critical Care Medicine, University of Pittsburgh School of Medicine, 3550 Terrace Street, Pittsburgh, PA, 15261, USA; 2 Halifax Health Medical Center, 303 N. Clyde Morris Blvd, Daytona Beach, FL, 32114, USA; 3 Intensive Care Unit, Department of Anesthesiology, Jikei University School of Medicine, 3-25-8, Nishi-Shimbashi, Minato-ku, Tokyo, 105-8461, Japan; 4 Department of Intensive Care and Department of Medicine, Austin Hospital and University of Melbourne, Studley Road, Heidelberg, Melbourne, 3084, Australia

## Abstract

**Introduction:**

Severe acute kidney injury (AKI) can be treated with either continuous renal replacement therapy (CRRT) or intermittent renal replacement therapy (IRRT). Limited evidence from existing studies does not support an outcome advantage of one modality versus the other, and most centers around the word use both modalities according to patient needs. However, cost estimates involve multiple factors that may not be generalizable to other sites, and, to date, only single-center cost studies have been performed. The aim of this study was to estimate the cost difference between CRRT and IRRT in the intensive care unit (ICU).

**Methods:**

We performed a *post hoc *analysis of a prospective observational study among 53 centers from 23 countries, from September 2000 to December 2001. We estimated costs based on staffing, as well as dialysate and replacement fluid, anticoagulation and extracorporeal circuit.

**Results:**

We found that the theoretic range of costs were from $3,629.80/day more with CRRT to $378.60/day more with IRRT. The median difference in cost between CRRT and IRRT was $289.60 (IQR 830.8-116.8) per day (greater with CRRT). Costs also varied greatly by region. Reducing replacement fluid volumes in CRRT to ≤ 25 ml/min (approximately 25 ml/kg/hr) would result in $67.20/day (23.2%) mean savings.

**Conclusions:**

Cost considerations with RRT are important and vary substantially among centers. We identified the relative impact of four cost domains (nurse staffing, fluid, anticoagulation, and extracorporeal circuit) on overall cost differences, and hospitals can look to these areas to reduce costs associated with RRT.

## Introduction

Renal replacement therapy (RRT) is one of the most common clinical procedures in the intensive care unit (ICU). Approximately 4-5% of critically ill patients require RRT during the ICU stay, a figure that is surprisingly consistent across countries [1]. However, the way in which RRT is provided varies greatly from one region to the next and even within regions or cities [2]. RRT can be classified into two major modalities: continuous RRT (CRRT) and intermittent RRT (IRRT). Although each modality has a different set of advantages and disadvantages [3-5], many patients may, at one time or another, be appropriate candidates for either therapy, especially when they are hemodynamically stable [5]. Results from randomized controlled trials and meta-analyses have failed to demonstrate a survival difference between these two modalities [6-12]. Thus, many authors have sought to determine whether any differences in costs exist when one modality is used instead of another [13,14].

Unfortunately, no multicenter study has been conducted to examine costs. Thus, the existing evidence is limited and poorly generalizable. Not surprisingly, costs are determined by labor (that is, provider staffing patterns) and materials (for example, fluids, anticoagulation, and dialyzers); these components vary widely across centers. As part of the B.E.S.T Kidney (Beginning and Ending Supportive Therapy for the Kidney) study, a multicenter, multinational, prospective, epidemiologic study aimed at understanding multiple aspects of RRT at an international level [1,15-17], data were obtained regarding each of these cost dimensions. Thus, as part of the larger study, which included patients from 53 centers and 23 countries, we sought to investigate the cost aspects of RRT practice across different centers in different countries around the world. Our aim was to determine the range and variation of costs across various centers and to provide a clear picture of the overall determinants of cost. Although costs at one center may bear little resemblance to those at another, the overall range of possible costs provides a meaningful metric whereby therapies can be compared.

## Materials and methods

### Subjects

This study was conducted at 53 centers in 23 countries, from September 2000 to December 2001. The study protocol was approved by the Investigational Review Board of the University of Pittsburgh as well as by the Ethics Committees or Investigational Review Boards of each participating site. Because of the anonymous and noninterventional nature of the study, Ethics Committees in most centers waived the need for informed consent. Where Ethics Committees or Investigational Review Boards required informed consent, we obtained formal written consent.

All patients who were older than 12 years (including seven patients younger than 18 years, because several units treated older children in their ICUs) and were admitted to one of the participating ICUs during the observational period were considered. From this population, we included only patients who were treated with RRT other than for drug poisoning. Patients with any dialysis treatment before admission to the ICU or patients with end-stage renal failure receiving chronic dialysis were excluded. For the current analysis, we considered all centers treating the patients described above but our analysis unit was the center not the patient--we included no patient-level data in this analysis.

### Measures

#### Data collection

Data were collected by means of an electronically prepared Excel-based data collection tool. This was made available to participating centers with instructions. All centers were asked to complete data entry and e-mail the data to the central office. On arrival, all data were screened in detail by a dedicated intensive care specialist for any missing information or logical errors or insufficient detail or any other queries. Any queries generated an immediate e-mail inquiry with planned resolution within 48 hours.

We divided the centers into six regions based on geographical area as follow: Northern Europe: Belgium, Czech Republic, Germany, Netherlands, Norway, Sweden, Switzerland, United Kingdom, and Russia; Southern Europe: Greece, Italy, Israel, Portugal, and Spain; North America: Canada, and the United States; South America: Brazil, and Uruguay; Asia: China, Indonesia, Japan, and Singapore; and Australia.

#### Cost analysis

All costs were converted to US dollars based on published exchange rates as of June 1, 2009.

Information on nursing assignments was available from all sites. Nursing time was determined by calculating the cost of additional nursing staff assigned to perform RRT or from the cost of changing ICU nurse staffing as a result of performing CRRT. Nursing cost was then determined from nursing time and from best available data from each center on hourly costs including all benefits. Where data were not available from the hospital itself, we used figures obtained from local nursing agencies. When no other source of data was available we estimated costs using data from similar institutions in the same region.

Dialysate and replacement fluid cost was calculated by multiplying the actual amount of fluid used for the first 24 hours by the cost of each type of fluid, which varied by each center and country. Because of the on-line dialysate production for IRRT, we only considered the cost of dialysate as coming from bicarbonate concentrate. Costs of replacement fluid for CRRT were calculated from each commercial supplier used by each institution. Data on fluid use was available from all sites, however the actual costs of each fluid was available from 50 sites. We did not consider costs associated with high volume hemofiltration, defined as replacement fluid rate more than 100 ml/min, in our analysis.

Anticoagulant cost was derived by multiplying the amount of anticoagulant used for the first 24 hours by the cost of anticoagulant. In most cases we obtained these costs directly from each site; when necessary we obtained the costs by contacting the manufacturer.

Extracorporeal circuit costs were estimated from the combined cost of the dialyzer and disposable blood lines which were used in the CRRT and IRRT systems. Data on dialyzer type was available from all sites, however the actual costs of each membrane was only available from 24 sites.

### Statistical Analysis

Due to the descriptive nature of our study we did not attempt to perform extensive statistical analysis. Cost differences for nursing cost, dialysate and fluid costs, anticoagulant costs, and extracorporeal circuit cost between CRRT and IRRT were calculated. The total range, the interquartile ranges (75% and 25%) and median values were calculated.

## Results

### Characteristic of organizational features

Organizational features of the 53 centers in 23 countries participating in our study are summarized in Table 1. Public hospitals composed the majority of sites, followed by private and mixed facilities. University-based hospitals were the most common, except in Australia, where large community hospitals predominated. Most participating centers contained between 500 and 999 beds. General (medical/surgical) ICU was the predominant type of ICU, and most of these contained between 10 and 19 ICU beds.

**Table 1:** Organizational features of RRT by regions

	Northern Europe	Southern Europe	North America	South America	Asia	Australia
1. Number of centers (%)	13 (24.5)	12 (22.6)	8 (15.1)	5 (9.4)	9 (17.0)	6 (11.3)

2. Number of countries (%)	9 (39.1)	5 (21.7)	2 (8.7)	2 (8.7)	4 (17.4)	1 (4.3)

3. Public or Private hospital						
- Public (%)	11 (84.6)	10 (83.3)	5 (62.5)	2 (40.0)	6 (66.7)	5 (83.3)
- Private %)	1 (7.7)	2 (16.7)	2 (25.0)	2 (40.0)	2 (22.2)	1 (16.7)
- Combine (%)	1 (7.7)	0	1 (12.5)	1 (20.0)	1 (11.1)	0

4. Type of hospital						
- University hospital (%)	10 (76.9)	6 (50)	8 (100)	2 (40.0)	7 (77.8)	2 (33.3)
- Large community (%)	3 (23.1)	3 (25)	0	2 (40.0)	2 (22.2)	4 (66.7)
- Small community (%)	0	3 (25)	0	1 (20.0)	0	0

5. Number of beds						
-499	2 (15.4)	4 (33.3)	0	2 (40.0)	1 (11.1)	4 (66.7)
-1499	5 (38.5)	5 (41.7)	6 (75.0)	3 (60.0)	4 (44.4)	2 (33.3)
- More than 999	6 (46.2)	3 (25)	2 (25.0)	0	4 (44.4)	0

6. Number of ICU beds						
-9	2 (15.4)	4 (33.3)	0	0	4 (44.4)	0
-29	6 (46.2)	8 (66.7)	0	0	4 (44.4)	5 (83.3)
-More than 19	5 (38.5)	0	8 (100)	8 (100)	1 (11.1)	1 (16.7)

7. Type of ICU						
- General/mixed	11 (84.6)	9 (75)	7 (87.5)	5 (100)	6 (66.7)	6 (100)
- Surgical	1 (7.7)	0	1 (12.5)	0	1(11.1)	0
- Specialty (Cardiothoracic, Bone marrow transplantation, etc.)	1 (7.7)	3 (25)	0	0	2 (22.2)	0

RRT, renal replacement therapy; ICU, intensive care unit.

### Physician and nursing practices

For IRRT in the ICU, we found that both intensivists and nephrologists prescribed therapy. However, at institutions where only one discipline prescribed, intensivists were responsible for prescribing more often than were nephrologists in Northern Europe (38.5% versus 23.5%) and in Asia (44.4% versus 22.2%), whereas nephrologists were the predominant prescribers in Southern Europe (33.3% versus 11.1%), North America (87.5% versus 12.5%), and South America.(100% versus none). For CRRT, intensivists prescribed therapy more than did nephrologists in Northern Europe (84.6% versus 7.7%), Southern Europe (41.7% versus 8.3%), Asia (88.9% versus 0%), and Australia (100% versus 0%), whereas nephrologists still played the major role in North America (62.5% versus 25%) and South America (80% versus 20%). In most regions, dialysis nurses cared for IRRT, whereas ICU nurses delivered CRRT (see Table 2).

**Table 2:** Treatment features of RRT by regions

	Northern Europe	Southern Europe	North America	South America	Asia	Australia
1. Who prescribes IRRT?						
- Nephrologist (%)	3 (23.5)	3 (33.3)	7 (87.5)	5 (100)	2 (22.2)	3 (60)
- Intensivist (%)	5 (38.5)	1 (11.1)	1 (12.5)	0	4 (44.4)	2 (40)
- Both (%)	5 (38.5)	5 (55.6)	0	0	3 (33.3)	0
						
2. Who prescribes CRRT?						
- Nephrologist (%)	1 (7.7)	1 (8.3)	5 (62.5)	4 (80)	0	0
- Intensivist (%)	11 (84.6)	5 (41.7)	2 (25)	1 (20)	8 (88.9)	6 (100)
- Both (%)	1 (7.7)	6 (50)	1 (12.5)	0	1 (11.1)	0
						
3. Who directs IRRT administration?						
- Physician (%)	0	0	0	0	1 (11.1)	0
- Dialysis nurse (%)	10 (76.9)	6 (75)	7 (87.5)	4 (80)	3 (33.3)	4 (80)
- ICU nurse (%)	1 (7.7)	2 (25)	1 (12.5)	0	3 (33.3)	1 (20)
- Technician (%)	1 (7.7)	0	0	0	2 (22.2)	0
- Physician and nurse	1 (7.7)	0	0	1 (20)	0	0
						
4. Who directs CRRT administration						
- Physician (%)	1 (7.7)	1 (9.1)	0	3 (60)	3 (33.3)	0
- Dialysis nurse (%)	2 (15.4)	2 (18.2)	4 (50)	1 (20)	0	0
- ICU nurse (%)	8 (61.5)	8 (72.7)	4 (50)	0	5 (55.6)	6 (100)
- Technician (%)	0	0	0	0	1 (11.1)	0
- Physician and nurse	2 (15.4)	0	0	1 (20)	0	0
						
5. Nurse-to-patient ratio for IRRT	1.3	1.1	1.5	1.1	1.3	1
						
6. Nurse-to-patient ratio for CRRT	1.2	1.7	1.4	1.2	1.4	0.8

RRT, renal replacement therapy; IRRT, intermittent renal replacement therapy; CRRT, continuous renal replacement therapy; ICU, intensive care unit.

### Nursing cost

**Figure 1:**
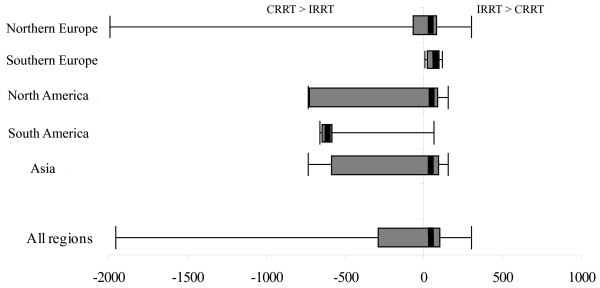
**Median difference and range of nursing costs by region**. The error bars represent the absolute range between the maximum nursing cost of CRRT and the minimum nursing cost of IRRT on the right, and between the maximum nursing cost of IRRT and minimum nursing cost of CRRT on the left. The box represents the 1^st ^and 3^rd ^quartiles of the nursing-cost range. The thick solid line represents the median difference in nursing costs for CRRT and IRRT across all centers in each region in which data were available.

We obtained nursing-cost data from 44 centers. Nursing costs were greater with IRRT in most regions ($25.70/day in Northern Europe, $47.10/day in Southern Europe, $38.60/day in North America, and $38.60/day in Asia (see Figure 1). The exception was Southern America, where CRRT is much more costly than IRRT ($681.40). In Australia, we cannot compare nursing costs for CRRT and IRRT because IRRT was not performed in the ICU at any of our sites.

### Dialysate and replacement fluid cost

**Figure 2:**
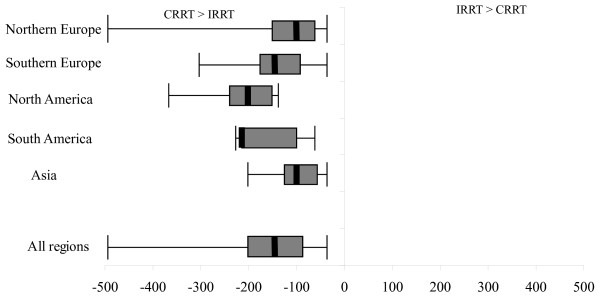
**Median difference and range of dialysate and replacement-fluid costs by region**. The error bars represent the absolute range between the maximum fluid cost of CRRT and the minimum fluid cost of IRRT, and between the maximum fluid cost of IRRT and minimum fluid cost of CRRT. The box represents the 1^st ^and 3^rd ^quartiles of the fluid-cost range. The thick solid line represents the median difference in fluid costs for CRRT and IRRT across all centers in each region in which data were available.

Given that dialysate can be compounded online by dialysis machines, fluid costs (available from 50 centers) were significantly greater with CRRT. South America was the region where the highest median difference of fluid cost was observed. Of note, the median treatment doses (combining dialysate and replacement fluid) for CRRT in each region were as follow: Northern Europe: 25.3 ml/min, Southern Europe: 25 ml/min, North America: 27.3 ml/min, South America: 33 ml/min, Asia was 21.3 ml/min, and Australia: 26.9 ml/min (Figure 2).

### Anticoagulant cost

**Figure 3:**
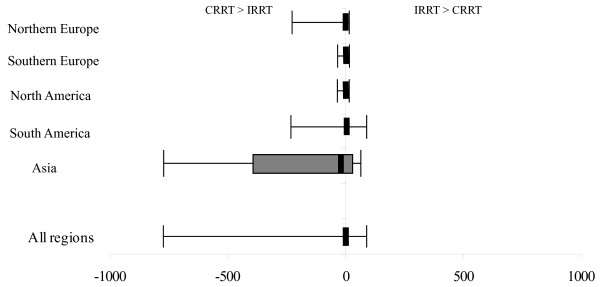
**Median difference and range of anticoagulant costs by region**. The error bars represent the absolute range between the maximum anticoagulant cost of CRRT and the minimum anticoagulant cost of IRRT, and between the maximum anticoagulant cost of IRRT and minimum anticoagulant cost of CRRT. The box represents the 1^st ^and 3^rd ^quartiles of the anticoagulant-cost range. The thick solid line represents the median difference in anticoagulant costs for CRRT and IRRT across all centers in each region in which data were available.

Anticoagulant costs were obtained from 49 centers. Heparin was the most commonly used anticoagulant for RRT, and overall, no significant difference was found for anticoagulant cost between IRRT and CRRT. The exception was Asia (specifically Japan), where anticoagulant costs for CRRT are significantly greater than for IRRT (see Figure 3).

### Extracorporeal circuit cost

**Figure 4:**
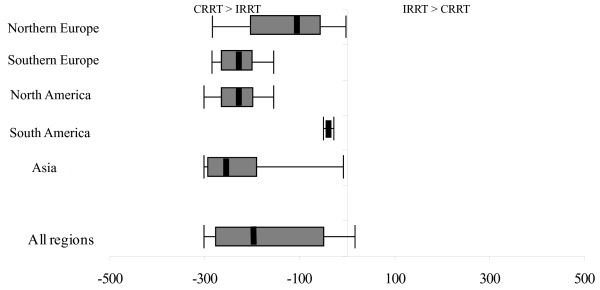
**Median difference and range of extracorporeal circuit costs by region**. The error bars represent the absolute range between the maximum extracorporeal circuit cost of CRRT and the minimum extracorporeal circuit cost of IRRT, and between the maximum extracorporeal circuit cost of IRRT and minimum extracorporeal circuit cost of CRRT. The box represents the 1^st ^and 3^rd ^quartiles of the extracorporeal circuit-cost range. The thick solid line represents the median difference in extracorporeal circuit costs for CRRT and IRRT across all centers in each region in which data were available.

The cost of extracorporeal circuits came from the blood lines and the dialyzers. Data, from 24 centers, show that for most regions, the costs of dialyzers were much greater than the costs of blood lines. Slightly different extracorporeal circuit costs were found between modalities. The region that demonstrated the most difference was Asia, followed by North America (Figure 4).

### Total cost

**Figure 5:**
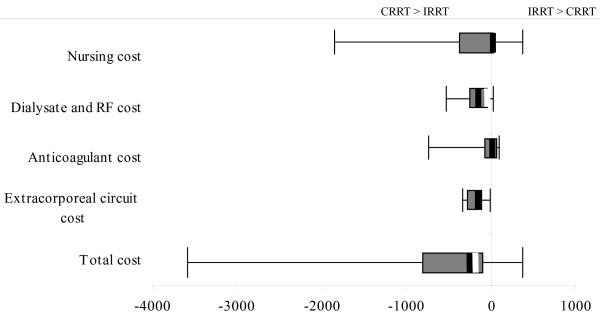
**Median difference and range of total cost by cost domain**. The error bars represent the range between the maximum cost of each domain for CRRT and the minimum cost for IRRT and the maximum cost of each domain for IRRT and minimum cost for CRRT. The box represents the 1^st ^and 3^rd ^quartiles of the total cost range. The thick solid line represents the range difference between the median cost differences for CRRT and IRRT. The thick white line represents the median difference of fluid costs when we limit replacement-fluid rate to 25 ml/min.

When we combined data from all regions, we found that dialysate and replacement fluid costs, and extracorporeal circuit costs, were generally greater for CRRT compared with IRRT. Furthermore, when combining all costs together (combined cost), we found that cost differences between CRRT and IRRT ranged from $3629.80/day more with CRRT to $378.60/day more with IRRT (Figure 5). A major contributor to cost differences between CRRT and IRRT was the cost of fluids. However, some of this cost reflected higher-volume CRRT (>25 ml/min) used at some sites. With ultrafiltration flow rates for CRRT of 25 ml/min (approximately 25 ml/kg/h), this could reduce fluid costs and combine cost by ~43.3% and 19.5%, respectively. We estimated the median cost difference between CRRT and IRRT across all centers to be $289.60/day (IQR, 830.80 - 116.8) per day (greater with CRRT). We calculated that reducing replacement-fluid volumes in CRRT to ≤ 25 ml/min would result in $67.20/day mean savings (23.2%).

## Discussion

This study is, to our knowledge, the first multicenter, multinational study that estimated cost differences between CRRT and IRRT in critically ill patients. We examined cost differences across four different domains and found significant variability in clinical practice. These differences resulted in a wide range of potential cost differences, ranging from greater costs with CRRT to greater costs with IRRT. In most regions, fluid and extracorporeal circuit costs were the largest contributors to the greater cost of CRRT.

Physician and nursing practice varied significantly by region. In North and South America, nephrologists were primarily responsible for both CRRT and IRRT, although intensivists in Northern Europe and Asia played a more dominant role for both therapies. For CRRT, we found that in Northern Europe, Southern Europe, Asia, and Australia, primarily intensivists prescribed CRRT. Our results are consistent with those of Ronco *et al*. [2], who reported survey data from 345 participants who attended two international meetings, and found that 35% of centers had only nephrologists, 18%, only intensivists, and 36% had both prescribing CRRT.

We found that the cost of CRRT was usually greater than that of IRRT, but this was not always so. Results from previous single- or two-center studies showed wide variability in cost estimates. Manns *et al*. [18] reviewed charts from two tertiary ICUs in Canada and demonstrated that the cost of performing CRRT ranged between Can $3,486/week and Can $5,117/week, whereas the cost of performing IRRT was Can $1,342/week. In the same year, Vitale *et al*. [19] reported the data from a single center in Italy, and found that the daily cost of CRRT was €276.70, whereas the daily cost of 4 h of IRRT was €247.83. Finally, Rauf *et al*. [20] estimated that mean adjusted costs through to hospital discharge were $93,611 and $140,733 among IRRT-treated and CRRT-treated patients, respectively. In our study, we found a range of total cost differences between CRRT and IRRT, which included these prior estimates but also included scenarios in which no difference in cost existed between the modalities, as well as scenarios in which IRRT was actually more expensive compared with CRRT.

Although our analysis included four separate cost domains, we could not estimate secondary cost differences arising from differences in resource allocation as a result of the different therapies. For example, CRRT may limit patient mobility to a greater extent compared with IRRT. If this difference resulted in greater use of physical therapists, additional secondary costs would be associated with CRRT. Conversely, if the use of CRRT were associated with improved renal recovery, as suggested by some observational studies [21], the added cost of continued renal support with IRRT would greatly increase cost differences in favor of CRRT. Available evidence from randomized trials has not demonstrated a survival benefit for CRRT when compared with IRRT [5,6,16-20]. Similarly, these trials have not found consistent differences in the ICU or hospital length of stay when one modality is used instead of the other. However, such head-to-head comparisons between IRRT and CRRT do not reflect clinical practice in most of the world where each modality is used to meet specific clinical needs [6]. Therefore, the portion of the RRT treatment that is considered to be discretionary between CRRT and IRRT may be limited. Nevertheless, it is important to note that cost differences between these modalities are determined largely by factors that can be modified.

For example, the cost of CRRT in our study was significantly influenced by the cost of fluids and therefore the rate of their use. When we limited effluent (replacement fluid plus dialysis) flow rate to 25 ml/min (~25 ml/kg/h), we could reduce fluid costs by ~43.3%. Given the results of the Acute Renal Failure Trials Network (ATN) study and the Randomized Evaluation of Normal versus Augmented Level (RENAL) Replacement Therapy Study [6,7], which found no survival advantage by increasing effluent flow rates to 35 and 40 ml/kg/h, respectively, reducing fluid use by reducing effluent flow rates to 25 ml/kg/h would seem prudent - provided that this minimal dose can be ensured.

Surprisingly, nursing staffing was a significant cost component of IRRT, as shown in Figure 5. This finding reflects two underlying practices that were highly variable across centers. First, some centers increased ICU nurse staffing (decreased nursing ratios) when CRRT was provided. In these centers, labor costs were greater with CRRT. By contrast, for centers providing 1:1 nursing for all ICU patients or not changing staffing when providing CRRT, labor costs can be greater only when IRRT requires additional staff from the dialysis unit. Second, given that most ICUs (as opposed to dialysis units) do not group their patients on dialysis, the typical IRRT session is delivered by a dedicated dialysis nurse. Thus, labor costs will inevitably be greater for IRRT relative to CRRT in centers where ICU nurse staffing does not change when CRRT is provided and when IRRT is provided by a dedicated (single-patient) dialysis nurse.

Another source of costs differences between CRRT and IRRT came from the use of anticoagulation. In Japan, the cost of anticoagulation is an important part of the total cost of RRT: nearly 50% of RRT patients (42.03%) in Japan were treated with nafamostat mesylate, a synthetic serine protease inhibitor that inhibits coagulation and fibrinolysis [22]. The cost of this drug is significantly greater than that of conventional heparin.

Our study had several limitations. First, it was not designed to estimate the fixed costs of RRT, such as the dialysis machine cost. Neither did we attempt to determine differences in physician billing, which varied depending on the health care system of each center and country.

Second, although we report a median cost difference between modalities among our centers, our primary goal was not to determine average costs. Instead, we intended to determine the range and variability of costs and their determinants. We believe that such information is more valuable to an individual practitioner or hospital, because local costs will vary but are likely to fall somewhere with the range we observed and are likely to be influenced by the same factors that we found in our study. Our median cost figure is undoubtedly a reflection of the composition of centers in our study, which may have been skewed toward those with a particular interest in AKI in the ICU. However, because we included a highly heterogeneous group of centers, the ranges of costs we report, as opposed to the point estimates, are likely to be highly generalizable.

Third, we had incomplete data on actual costs for certain domains and used regional references to estimate these costs. These regional references likely underestimate the variability between centers, particularly in some regions.

Finally, we accepted that a mixture of developed and developing countries exists in some regions such as in Asia. Furthermore, our categorization of countries by region was somewhat arbitrary, and wide differences may exist between practice patterns within each region. However, when the primary analysis is repeated after excluding the 44 patients from three centers in countries with arguably very different healthcare delivery systems (14 patients from Russia, six patients from China, and 24 patients from Indonesia), our results were not materially changed. We also realize that we may underestimate the cost of anticoagulation, because we do not include the cost of monitoring of anticoagulation such as ionized calcium, or aPTT/ACT. However, our intent was to provide an overall picture of the range of cost differences between IRRT and CRRT, rather than specifically to estimate costs in each region. Thus, the cost landscape we were able to illustrate provides the first international glimpse into this important area.

## Conclusions

Cost considerations with RRT are important and vary substantially among centers. Major contributors to RRT costs included nurse staffing, dialysate and replacement fluid, anticoagulation, and extracorporeal circuit costs. We found that the confidence intervals for cost differences between CRRT and IRRT were wide and crossed zero. Therefore, single-center cost estimates will lack generalizability. We identified the relative impact of four cost domains on overall cost differences, and hospitals can look to these areas to reduce costs associated with RRT. Reducing effluent flow rates to 25 ml/min (~25 ml/kg/h) has the capacity to reduce fluid costs and combined costs by ~43.3%, and 19.5%, respectively.

## Key messages

• Combined cost differences across four domains (nursing staff, fluid, anticoagulation, and extracorporeal circuit cost) of CRRT are higher than those of IRRT.

• Cost differences are highly variable across centers and include scenarios in which either therapy is more or less expensive compared with the other.

• Fluid and extracorporeal circuit costs are major determinants of cost for CRRT, whereas human resource costs (nursing) are the major determinant of cost for IRRT.

• Limiting the rate of replacement fluid to 25 ml/min, as per the current best evidence for dose of CRRT, can reduce the fluid cost and combined cost of CRRT and the median difference in cost between CRRT and IRRT by ~43.3%, 19.5%, and 23.2%, respectively.

## Abbreviations

AKI: acute kidney injury; BEST Kidney: Beginning and Ending Supportive Therapy for the Kidney; CRRT: continuous renal replacement therapy; ICU: intensive care unit; IQR: interquartile range; IRRT: intermittent renal replacement therapy; RRT: renal replacement therapy.

## Competing interests

JK and RB received funding and consulting fees from companies that make dialysis equipment and supplies (Gambro, Baxter, Fresenius). No company financed the current work or has any role in the content.

## Authors' contributions

NS analyzed data and wrote and revised the manuscript. LL analyzed data. SU collected data, developed the study protocol, and revised the manuscript. RB collected data, developed the study protocol, and revised the manuscript. JK collected data, developed the study protocol, and revised the manuscript. All authors read and approved the final manuscript.
